
JOURNAL INFORMATION
==============================
NLM Title Abbreviation: Wirtschaftsdienst
Journal ID: Wirtschaftsdienst
NLM Journal ID: 101086612

Wirtschaftsdienst (Hamburg, Germany : 1949)

ISSN: 0043-6275

ARTICLE INFORMATION
==============================
PMCID: PMC8218274
PMID: 34176983
DOI: 10.1007/s10273-021-2927-0
Article ID: 2927
Article version: 1
Subjects: Leitartikel

Wie kann die weltweite Impfstoffproduktion gesteigert werden?

How can Global Vaccine Production be Increased?

Licht Georg Georg.Licht@zew.de

Wambach Achim achim.wambach@zew.de

grid.13414.33 0000 0004 0492 4665 ZEW, L 7, 1, 68161 Mannheim, Deutschland
Electronic publication date: 2021 Jun 22
Print publication date: 2021
Volume: 101
Issue: 6
First page: 406
Last page: 407
Copyright: © Der/die Autor:in(nen) 2021
License: Open Access: Dieser Artikel wird unter der Creative Commons Namensnennung 4.0 International Lizenz veröffentlicht (https://creativecommons.org/licenses/by/4.0/deed.de).
Open Access wird durch die ZBW — Leibniz-Informationszentrum Wirtschaft gefördert.
License URL: https://creativecommons.org/licenses/by/4.0/

issue-copyright-statement: © The Author(s) 2021

==============================

Tedros Adhanom Ghebreyesus, der Generaldirektor der Weltgesundheitsorganisation, fand drastische Worte anlässlich der Jahresversammlung der Gesundheitsminister der 194 Mitgliedstaaten im Mai: Die COVID-19-Pandemie werde durch die skandalöse Ungleichheit bei der Verteilung der Impfstoffe aufrechterhalten. 75 % aller bislang verabreichten Impfungen erfolgten in nur zehn Ländern. Ziel müsse es sein, die internationale Initiative COVAX, die bislang lediglich für 1 % der Menschen in den von ihr berücksichtigten 124 Ländern Impfstoffe bereitstellen konnte, bis Jahresende mit Impfstoffen für 30 % der Menschen zu versorgen. Neben einer anderen Verteilung der existierenden Impfstoffe ist dafür vor allem eine massive Ausweitung der Produktion von Impfstoffen nötig. Ein Vorschlag, um dies zu erreichen, der auch von US-Präsident Joe Biden unterstützt wird, ist, die Patente für Corona-Vakzine freizugeben. Der Vorschlag greift eine Initiative von Südafrika und Indien vom Oktober 2020 in der WTO auf, die eine zeitweilige Aussetzung des Patentschutzes für alle Produkte, die zur Vorbeugung, Eindämmung und Behandlung von COVID-19 notwendig sind, vorsah (Patent Waiver).

Die Befürwortenden argumentieren, dass Patentrechte und die damit verbundene exklusive Nutzung durch die jeweiligen Pharmaunternehmen den Ausbau der Impfstoffproduktion bremsen. Die Gegner:innen der Aussetzung des Patentschutzes betonen, dass der Auf- und Ausbau der Produktionskapazitäten Zeit und spezifisches Know-how benötige. Patentrechte stellen dabei nicht den begrenzenden Faktor dar. Welches Argument wiegt schwerer? Klar ist: Zur Diskussion steht das Geschäftsmodell der pharmazeutischen Industrie. Erforschung und Entwicklung neuer Impfstoffe und Medikamente sind langwierig, riskant und teuer. Erfolgreichen Unternehmen winkt im Gegenzug ein zeitlich begrenztes Monopol für Produkt- und Prozessinnovationen. Nicht erfolgreiche Unternehmen bleiben auf ihren Kosten sitzen. Das vorübergehende Monopol ermöglicht den erfolgreichen Unternehmen laut den Standardlehrbüchern der Ökonomie, vergleichsweise hohe Preise zu verlangen. Die anfänglichen Investitionen werden nur in Erwartung dieser Monopolposition getätigt. Alerdings führen zu hohe Monopolpreise dazu, dass Impfstoffe oder Medikamente für viele Menschen unbezahlbar bleiben. Aber trifft dieses Standardlehrbuchmodell auf die aktuelle Situation überhaupt zu? Die Aussicht auf hohe Gewinne und staatliche Unterstützung führte im Fall von COVID-19 zur Entwicklung mehrerer Impfstoffe, die auf unterschiedlichen Technologien beruhen – und damit dem Bild des einen Monopolisten widersprechen. AstraZeneca und Johnson&Johnson verwenden die etablierte Technologie der Vektor-Impfstoffe, Moderna, Biontech/Pfizer und Curevac greifen auf die von ihnen maßgeblich entwickelte mRNA-Technologie zurück, die nun erstmals auf breiter Front angewendet wird, und schließlich erwarten Novavax sowie GSK/Sanofi bis zum Jahresende die Zulassung ihrer klassischen, protein-basierten Impfstoffe. Zudem haben Unternehmen aus China, Indien und Russland eigene Impfstoffe entwickelt. Mit Blick auf den angestrebten Ausbau der Produktionskapazitäten gibt es zwischen den Herstellern der Impfstoffe also bereits einen gewissen Wettbewerb. Hinzu kommt, dass die Pharmaunternehmen für ihre Impfstoffe nicht einfach wie ein Lehrbuchmonopolist Preise setzen, sondern diese mit den jeweiligen Ländern aushandeln. Die Macht der Monopolisten ist mithin beschränkt.

Das Geschäftsmodell der pharmazeutischen Industrie erwies sich also als sehr erfolgreich darin, in historisch kurzer Zeit technologisch unterschiedliche Lösungen für wirksame Impfstoffe zu entwickeln. Eine Aussetzung des Patentrechts hätte schwer abzuschätzende Folgen für in Zukunft notwendige pharmazeutische Innovationen. Daher sind alternative Lösungen zur Steigerung der Impfstoffproduktion gefragt. Solche Vorschläge gibt es bereits: So könnte das Budget von COVAX aufgestockt werden. COVAX würde die zusätzlichen Mittel nicht nur zum Kauf von Impfstoffen nutzen, sondern auch zum Aufbau von Produktionskapazitäten. Ein weiterer Vorschlag läuft darauf hinaus, nationale Impfstoff-Egoismen zu überwinden, sprich auf Exportstopps für Impfstoffe und wichtige Vorprodukte zu verzichten. Eine solche Öffnung der Märkte könnte die COVAX-Initiative ergänzen. Mit einem Ausbau der Produktionsstätten für die Impfstoffe ist es aber nicht getan. Die Gefahr vielfältiger Virusvarianten ist real, auch und gerade weil erst so wenige Menschen weltweit geimpft sind, und es wird entscheidend sein, weitere Forschung an Corona-Impfstoffen und -Medikamenten voranzutreiben. Hinzu kommt, dass die verfügbaren Vakzine wohl schon sechs Monate nach der Injektion an Wirksamkeit einbüßen. Auffrischungsimpfungen werden nötig und damit bleibt der Bedarf absehbar hoch. Die Forschung an neuen Impfstoffen, die eine längere Immunisierung versprechen, wäre ein wichtiger Beitrag, um zu verhindern, dass – einhergehend mit neuen Virusvarianten – Corona in den kommenden Jahren erneut aufflammt. Schnelle Forschungsergebnisse können erreicht werden, wenn ungeachtet bestehender oder zukünftiger Patente Forschende aller Länder vorbehaltlos und offen miteinander zusammenarbeiten. Es gibt nicht das eine Patent, sondern eine Vielzahl sich (partiell) ergänzender patentierter Innovationen, sei es bezüglich des Impfstoffs, seiner Komponenten, der Herstellungsweise von Zwischensubstanzen und Endprodukten, der Produktformulierung und -verabreichung. Bereits vor einigen Jahren beschrieben Forschende die Gefahr, dass die Unsicherheit über das Bestehen und die Durchsetzungsfähigkeit von Patenten an den erforschten Gegenständen die Investitionsneigung hemme.

Bislang sind komplizierte Übereinkommen zwischen Nutzenden und Inhabenden von Patenten notwendig, damit patentgeschützte Produkte, Herstellungsverfahren oder Forschungswerkzeuge verwendet werden können. Ein funktionierender COVID-19-Patentpool könnte sowohl die frühzeitige Offenlegung und Nutzung der Patente stimulieren als auch die Lizenzvergabe über One-Stop-Shop verbunden mit einer Standardisierung vereinfachen und beschleunigen. Dem COVID-19 Technology Access Pool (C-TAP) der WHO blieb jedoch bislang der Erfolg versagt, da lediglich an die „globale Verantwortung“ der Patentrechteinhabenden appelliert wurde. Gleichwohl wäre ein solcher Patentpool eine Möglichkeit, im Rahmen des bestehenden Patentsystems die Nutzungsmöglichkeiten zu erweitern und die Wissenszirkulation zu beschleunigen. COVAX könnte einen Beitrag leisten und zur notwendigen Finanzierung von C-TAP beitragen. Die Pharmaindustrie scheint sich ihrer Verantwortung bewusst zu sein: So ist es zu begrüßen, dass Moderna bereits im Oktober ankündigte, dass es seine „COVID-19-bezogenen Patente nicht gegen diejenigen durchsetzen wird, die [andere] Impfstoffe zur Bekämpfung der Pandemie herstellen“.

Ein Beitrag zur COVID-19-Forschung könnte auch durch eine Freigabe der Patente für Forschungszwecke erreicht werden. Zwar kennen viele Patentgesetze ein Forschungsprivileg – wie das deutsche Patentgesetz in § 11, Nr. 2 – und ermöglichen damit den Einsatz von Patenten zur Weiterentwicklung oder zum Test der Tauglichkeit eines patentierten Verfahrens. Allerdings umschließt das nicht den Einsatz patentgeschützter Erfindungen als Werkzeuge zur Entwicklung neuartiger Verfahren. In einer arbeitsteiligen Forschung stellt das Endprodukt des einen Unternehmens das Forschungswerkzeug eines zweiten Unternehmens dar. Der Grat zwischen erlaubter Nutzung und Patentverletzung ist schmal. Die Implementation des Forschungsprivilegs in den nationalen Patentgesetzen kennt zudem große Unterschiede. Eine höhere Klarheit könnte damit einen Beitrag zu Stärkung und Beschleunigung der Forschung für neue COVID-19-Impfstoffe und -Medikamente liefern. Keine Frage: Auch eine Außerkraftsetzung der Patentrechte für Forschungszwecke läuft auf eine Einschränkung der Eigentumsrechte betroffener Pharmaunternehmen hinaus und ist hinsichtlich der Anreizwirkung für künftige Forschung nicht unproblematisch. Dennoch könnte dieses Instrument angesichts der weltweiten Bedrohung durch neue Virusvarianten in der jetzigen Situation angemessen sein. Zumal von der Außerkraftsetzung der Patentrechte auch bisherige Inhabende insofern profitieren würden, da sie für Forschungszwecke auf die Patente der anderen Impfstoffhersteller zugreifen könnten.

Georg Licht
ZEW – Leibniz-Zentrum für Europäische Wirtschaftsforschung
georg.licht@zew.de

Achim Wambach
ZEW – Leibniz-Zentrum für Europäische Wirtschaftsforschung
achim.wambach@zew.de

Dr. Georg Licht leitet den Forschungsbereich Innovationsökonomik und Unternehmensdynamik am ZEW.

Prof. Achim Wambach, Ph. D., ist Präsident des ZEW — Leibniz-Zentrums für Europäische Wirtschaftsforschung in Mannheim.

